# DESIGNING FAMILY CAREGIVER STUDIES THAT BALANCE STRESS PROCESS AND HELPING RELATIONSHIP PERSPECTIVES

**DOI:** 10.1093/geroni/igz038.873

**Published:** 2019-11-08

**Authors:** David L Roth, William Haley, Orla Sheehan, Jeremy Walston, David Rhodes, Virginia Howard

**Affiliations:** 1 Johns Hopkins University School of Medicine, Baltimore, Maryland, United States; 2 University of South Florida, Tampa, Florida, United States; 3 Johns Hopkins University, Baltimore, Maryland, United States; 4 University of Alabama at Birmingham, Birmingham, Alabama, United States

## Abstract

Family caregiving is often characterized as a chronically stressful situation, and stress process models have been the dominant conceptual foundation underlying caregiving studies for decades. Recently, this perspective has been augmented with more positive views that emphasize potentially healthy and prosocial aspects of caregiving. Replicated findings from population-based studies show that caregivers have lower mortality rates than noncaregivers, consistent with the more balanced conceptual approach. The Caregiving Transitions Study is investigating 251 participants who transitioned into a caregiving role at some point between two blood samples taken 10 years apart in a national epidemiological study and 251 matched controls. Preliminary analyses confirm that caregiving leads to increased psychological distress. Ongoing analyses are examining changes in inflammatory biomarkers, health status, and positive aspects of caregiving. Findings will be examined alongside our recent meta-analysis of convenience samples that found caregiving to have small and inconsistent relationships with biomarkers of inflammation and immunity.

